# Moyamoya syndrome secondary to mitochondrial disease in a patient with partial trisomy 13q14 and 13q31: A novel case report and literature review

**DOI:** 10.1016/j.heliyon.2023.e13466

**Published:** 2023-02-04

**Authors:** Saleha Abdul Rab, Tarek Ziad Arabi, Hiba Muhammad Raheel, Belal Nedal Sabbah, Nowar Habib Zain AlAbidien, Abdulaziz Alsemari

**Affiliations:** a College of Medicine, Alfaisal University, Riyadh, Saudi Arabia; b Department of Neuroscience, King Faisal Specialist Hospital and Research Centre, Riyadh, Saudi Arabia

**Keywords:** Moyamoya syndrome, Patau syndrome (trisomy 13), Partial trisomy 13q, Pediatric stroke, Mitochondrial disease, Epilepsy

## Abstract

Moyamoya syndrome (MMS) is a cerebrovascular disease characterized by stenosis of the internal carotid arteries and the formation of an abnormal vascular network at the base of the brain. MMS usually occurs secondary to various conditions, particularly Down syndrome, and sickle cell anemia, and presents with motor deficits, sensory symptoms, recurrent ischemic strokes, hemodynamic transient ischemic attacks, recurrent seizures, and hemorrhage. Trisomy 13 (Patau Syndrome) is a chromosomal abnormality that may be characterized by full or partial trisomy of chromosome 13. Phenotypic features of partial trisomy 13 include leukoencephalopathy, hippocampal hypoplasia, intellectual disability, facial anomalies, and others. Herein, we report a case of a 19-year-old female diagnosed with partial trisomy 13q, characterized by two large duplications in the 13q14 and 13q31 regions, with trisomy-induced bilateral MMS – the first known case to be discussed in literature. Particularly, our patient was identified to have a gain of 22Mb within the 13q14.11q21.31 region – a duplication that has not been described previously. Our patient suffered four strokes between the ages of 5 and 7, later developing intractable seizures, hemiplegia, spasticity in all limbs, global delay, and regression. Despite bilateral encephaloduroarteriosynangiosis and being on several antiepileptic medications, the MMS continued to progress, confounded by the partial trisomy 13. Studies must elucidate the association between mitochondrial damage and MMS, as well as mechanisms of epilepsy associated with chromosomal abnormalities, particularly in the context of underlying mitochondrial diseases.

## 1 Introduction

Moyamoya syndrome (MMS) is a cerebrovascular disease characterized by stenosis of the internal carotid arteries (ICA) and formation of an abnormal vascular network at the base of the brain [1]. MMS is a term coined for moyamoya vasculopathy in patients with an underlying cause and is usually unilateral [2,3]. MMS has been reported secondary to various conditions, most notably neurofibromatosis type 1, Down syndrome, thyroid disease, and sickle cell anemia [4]. The clinical manifestations of MMS include recurrent ischemic strokes, hemodynamic transient ischemic attacks (TIAs), recurrent seizures, and hemorrhage. A variety of other symptoms may also be present, including motor deficits, sensory symptoms, speech disturbances, and headaches [5].

Trisomy 13 (Patau Syndrome) is an uncommon and severe chromosomal abnormality caused by a trisomy of chromosome 13 and is associated with a prevalence rate of 1.68/10,000 [6]. Patau Syndrome may be characterized by full or partial trisomy. While the exact frequency of partial trisomy 13q is yet to be determined, it is estimated to account for <1% of all cases of Trisomy 13 [7,8]. Possible mechanisms leading to partial trisomy 13q include parental reciprocal translocations, parental pericentric inversions, or de novo direct duplications [8]. Phenotypic features include leukoencephalopathy, epilepsy, stroke, hippocampal hypoplasia, intellectual disability, facial anomalies, olfactory hypoplasia, and cardiomyopathy [9,10].

The mechanisms leading to the characteristic symptoms of trisomy 13 are not yet well defined. However, generation of reactive oxidative species (ROS) seems to play a key role in its pathogenicity [11]. Studies have reported that the generated ROS leads to mitochondrial dysfunction [11]. Specifically, mitochondrial dysfunction is implicated in the pathogenesis of moyamoya disease/syndrome, with one study demonstrating that the endothelial colony-forming cells (ECFCs) of patients with moyamoya carried morphologically and functionally abnormal mitochondria [12].

Mitochondrial abnormalities are also implicated in neuronal damage, as neurons are mostly dependent on mitochondria in oxidative stress conditions [11]. These mitochondrial diseases are defined as genetic disorders that result in a primary defect in mitochondrial oxidative phosphorylation [13]. Particularly, errors in mitochondrial DNA (mtDNA) or nuclear DNA (nDNA) can lead to respiratory chain defects, rendering the mitochondria unable to generate enough energy to supply the needs of various organs [14]. This energy deficiency results in mitochondrial proliferation within the smooth muscle and the endothelial cells of small blood vessels, causing angiopathy and impaired perfusion to the body organs, eventually culminating as multi-organ failure. One example of such an illness is mitochondrial encephalomyopathy, lactic acidosis, and stroke-like episodes (MELAS) syndrome [15].

Herein, we report a case of a 19-year-old female diagnosed with partial trisomy 13q, characterized by two large duplications in the 13q14 and 13q31 regions, with trisomy-induced mitochondrial dysfunction, presenting with bilateral MMS. To the authors’ knowledge, this is the first article reporting an association of trisomy 13 with MMS. This case report is reported in accordance with the CARE guidelines [16].

## 2 Case presentation

**Fig. 1:**
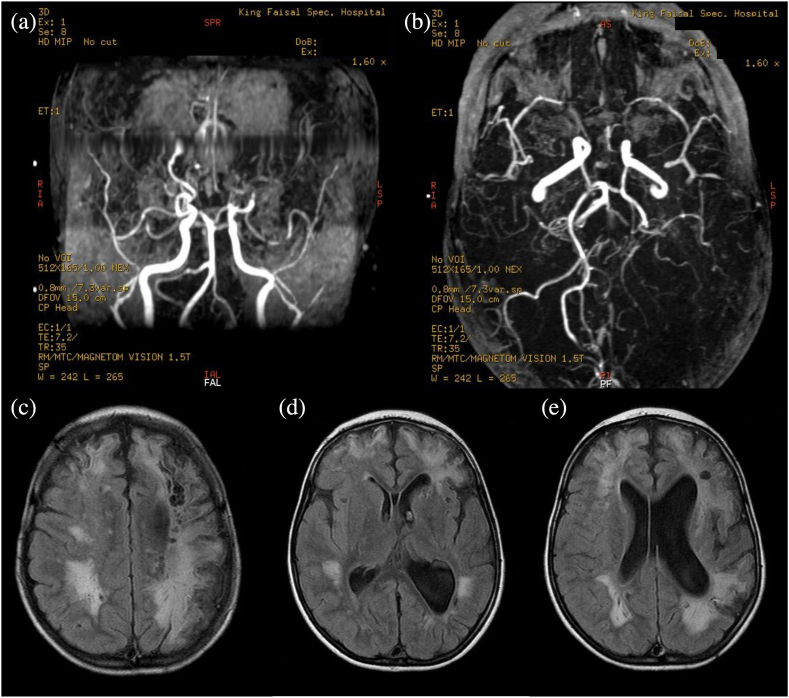
The MRA of the intracranial circulation on (a) coronal, and (b) axial view, showing significant bilateral supraclinoid ICA loss of normal flow, with poor flow in the middle cerebral arteries and the anterior cerebral arteries bilaterally. Prominent collateral flow is also detected in the perforator branches. These findings are consistent with a moyamoya syndrome pattern. Posterior circulation shows good flow in the basilar artery as well as in the posterior cerebral arteries on both sides. FLAIR MRI taken (c) below the level of the caudate nucleus, (d) at the level of the caudate nucleus and genu of the corpus collosum, and (e) at the level of the occipital horns of the lateral ventricles, showing multiple infractions in both hemispheres. Significant cystic encephalomalacic changes are seen involving the supratentorial brain bilaterally, with more involvement of the left hemisphere. The frontal and parietal areas are also involved. Periventricular hyperintensity is detected, especially in the parietal regions on both sides, most likely representative of small vessel ischemic changes (however, associated white matter disease cannot be completely excluded). There is significant prominence in the perivascular spaces, with multiple well-defined lacunar infarcts in the basal ganglia, especially in the left caudate head. Ex vacuo enlargement of the left lateral ventricle is present, related to the diffuse ischemic changes and volume loss. Thinning of the corpus callosum is present related to the overall brain atrophy and ischemic changes. These findings are most likely secondary to vascular events, especially with the significant vascular abnormalities consistent with moyamoya type pattern.

Our patient, a young Saudi female, is the youngest child of non-consanguineous parents, and has two older siblings who are healthy. She first presented to our hospital at the age of 6 years with a history of bilateral frontal stroke and left partial stroke. Her first stroke occurred when she was less than 6 years old, presenting with right-sided weakness and seizures. The seizures began as absence seizures and eventually progressed to tonic seizures. The patient was managed with antiepileptic drugs and blood thinners. Radiology did not reveal arteriovenous malformation or aneurysm. The patient had her second stroke a few months later, which resulted in more severe right-sided weakness. Moyamoya syndrome was suspected due to the recurrent cerebrovascular accidents, and a magnetic resonance angiography (MRA) (Fig. 1a and b) and brain magnetic resonance imaging (MRI) (Fig. 1c, d, 1e) confirmed the diagnosis.

Left-sided encephaloduroarteriosynangiosis (EDAS) was performed three months later, but the patient's seizure activity persisted. Thus, she was diagnosed with epilepsy, and was started on prescription antiepileptic drugs, namely valproic acid, clobazam, and levetiracetam.

**Fig. 2:**
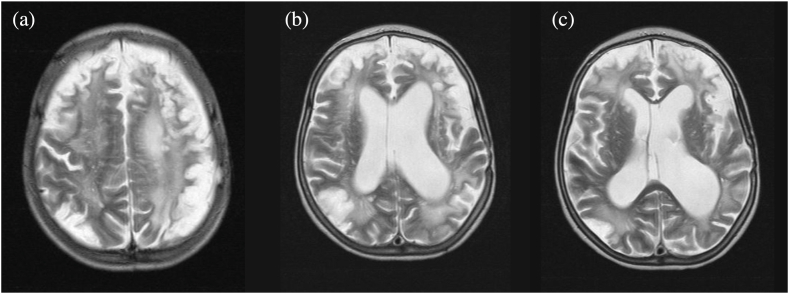
T2-weighted MRI taken(a) below the level of the caudate nucleus, (b) at the level of the caudate nucleus and genu of the corpus collosum, and (c) at the level of the occipital horns of the lateral ventricles, after the patient presented with her third stroke. This MRI shows subacute infarction in almost the entire right temporal lobe corresponding to MCA territory, with increased diffusion without significant mass effect. There are similar findings in the cortical gray matter anteriorly in left temporal lobe with increased diffusion. There are severe old ischemic lesions bilaterally, more on the left side, which are unchanged since the previous study with secondary enlargement of the ventricular system supratentorially. The posterior fossa is grossly unremarkable.

The patient was then brought to our hospital, where she was scheduled for right-sided EDAS. However, she suffered a third stroke involving the right middle cerebral artery while awaiting the procedure (Fig. 2a–c). MRA from the same time also showed reduced number of arteries with flow voids in the sylvian fissures bilaterally as compared to the previous MRI. Hence, the decision was made to postpone EDAS for 2–3 months to give the patient time to recover. One month later, before EDAS could be performed, the patient had her fourth stroke, presenting with lethargy and poor feeding for the past 5 days, as well as increased seizure activity. It was this event that left the patient completely bedridden, non-verbal, and dependent. She developed flaccid right-sided hemiparesis with exaggerated reflexes, severe left-sided weakness, continuous drooling, and dysphagia. Right-sided EDAS was performed one month after her fourth stroke. The procedure involved creating an extracranial to intracranial bypass using a superficial temporal artery graft.

Despite undergoing EDAS on both sides, the patient's condition did not improve, and she continued experiencing intractable seizures. She was continued on valproic acid, levetiracetam, and clobazam. The patient developed bilateral facial palsy and was no longer able to tolerate oral intake without risk of aspiration. At the age of 7, Nissen fundoplication and gastrostomy were performed five months after right-sided EDAS to allow easier feeding.

By the time the patient was 14 years old, she had developed global developmental delay and regression, uncontrollable seizures, and was on multiple antiepileptic medications. Brain MRI performed at age 14 showed scattered old ischemic insults with laminar necrosis, gross parenchymal loss, and gliotic changes with ex vacuo dilatation of the ventricles. No acute intracranial hemorrhage or acute infarction was seen. Further investigations revealed uncorrectable hyponatremia (related to her neurological status), constipation, precocious puberty, and scoliosis. Correction of hyponatremia to the level of no seizure activity became the aim of treatment, however, did not prove to be successful.

Additionally. high lactate levels were detected in the venous blood gases of the patient. Thus, magnetic resonance spectroscopy was performed at an external hospital, and small lactate peaks were identified in the basal ganglia regions. Unfortunately, this image could not be retrieved currently for the purpose of this case report. However, given the patient's history of recurrent strokes, this led us to suspect mitochondrial encephalomyopathy, lactic acidosis, and stroke-like episodes (MELAS) syndrome. Comparative genomic hybridization (CGH) was performed to rule out this condition and revealed partial Trisomy 13.

Whole exome sequencing (WES) performed when the patient was 17 years old revealed several mitochondrial diseases among others, seen in Table 1.

**Table 1:** Whole exome sequencing, performed at age 17, revealing several mitochondrial diseases. The OMIM number, zygosity, mode of inheritance, and variants are listed for each, as per the report.

Phenotype(s)	OMIM No.	Zygosity	Inheritance	Variant
Mitochondrial complex I deficiency, nuclear type 24 (MC1DN24)	618245	Homozygous	Autosomal recessive	NDUFB9: NM_001278645: exon3: c.139T > G: p.C47G
Mitochondrial complex I deficiency, nuclear type 16 (MC1DN16)	618238	Heterozygous	Autosomal recessive	NDUFAF5: NM_001039375: exon6: c.509G > A: p.R170Q
Epilepsy, generalized, with febrile seizures plus, type 2	607208	Heterozygous	Autosomal dominant	SCN1A: NM_001165963: exon26: c.5501C > T: p.A1834V
Mitochondrial DNA depletion syndrome 13 (encephalomyopathic type)	615471	Heterozygous	Autosomal recessive	FBXL4: NM_012160: exon3: c.65G > C: p.R22P

Chromosomal microarray analysis (CMA) performed when the patient was 19 years old identified two large duplications that were consistent with a genetic diagnosis of partial 13q trisomy (Table 2)

1. A gain of 22Mb within the 13q14.11q21.31 chromosomal region, determined to be likely pathogenic, non-distal trisomy 13q: The proximal long arm of chromosome 13 is partially duplicated, resulting in a highly variable phenotype. While the exact nature of this duplication has never been described in literature, similar duplications have been related to partial trisomy 13q in the past. Growth and developmental delays accompany this phenotype, as well as craniofacial dysmorphisms [17].
2. A gain of 24Mb within the 13q31.3q34 chromosomal region, determined to be pathogenic, distal trisomy 13q: In distal trisomy 13q, the distal long arm of chromosome 13 is partially duplicated, again resulting in a variable phenotype. In addition to microcephaly and holoprosencephaly (incomplete development of the forebrain), this phenotype may be characterized by intellectual disability, psychomotor delay, and craniofacial dysmorphism [18].

**Table 2:** Results of the chromosomal microarray analysis, performed at age 19, confirming the genetic diagnosis of partial 13q trisomy. The duplications, as well as their interpretations, are listed as per the report by Centogene®. Interpretation of cytogenetic results (Table 2) as per the report by Centogene®.

Duplication	Interpretation	Related Disorder
A gain of 22Mb within the 13q14.11q21.31 chromosomal region	Likely pathogenic	Non-distal trisomy 13q
A gain of 24Mb within the 13q31.3q34 chromosomal region	Pathogenic	Distal trisomy 13q

**Fig. 3:**
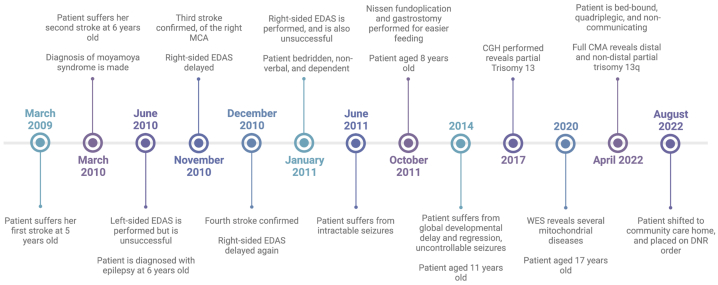
A graphic timeline summarizing the chronologic order of events that took place in our case report.

All WES and CMA data from this patient can be found on a dataset on **Mendeley Data, V1**, doi: 10.17632/7b8nf7gp3x.1 [19]. A full timeline of the patient's clinical history can be found in Fig. 3. The patient is now 19 years old and continues to suffer refractory seizures. She is bed-bound, spastic in all limbs, quadriplegic, and non-communicating. She continues to feed through a gastrostomy tube. For this reason, she is susceptible to gastrointestinal issues caused by unclean feed administration or change in feed and frequently has gastrointestinal complaints, such as vomiting and diarrhea. She has undergone several admissions for recurring pneumonia, likely due to aspiration of fluid contents secondary to vomiting. The patient is continued on phenobarbital, valproic acid, levetiracetam, and clobazam, and is currently under Do Not Resuscitate (DNR) orders at a community care home.

## 3 Discussion

MMS is a cerebrovascular condition characterized by stenosis of the ICAs causing recurrent strokes, TIAs, and seizures [5]. It has been established that MMS occurs to pre-existing conditions such as neurofibromatosis type 1, trisomy 21, thyroid disorders, and cranial irradiation [4], and is defined by the unilateral ICA involvement [3].

MMS is a widely recognized cause of stroke in children and must be considered among the differential diagnoses of any child presenting with symptoms of cerebral ischemia. It can be identified on brain CT or MRI by the visualization of small collateral vessels growing in the bottom of the cerebrum, resembling a “puff of smoke”. A CT scan may also reveal hemorrhage and atrophy of the affected hemisphere [20]. Early operative intervention is recommended to prevent progression of the condition and development of complications. A surgical treatment known as EDAS is performed, which involves the transposition of an artery taken from the scalp onto the surface of the brain [21]. EDAS aims to improve collateral blood flow in the brain and has been proven to be successful in the treatment of MMS [21].

Trisomy 13, or Patau Syndrome, is a chromosomal aberration that results in an extra duplicate of chromosome 13 being present in the body cells. Trisomy 13 may be full trisomy 13 (a third copy of chromosome 13 exists in all cells), mosaic trisomy 13 (a third copy of chromosome 13 exists only in some cells), or partial trisomy 13 (only a partial third copy of chromosome 13 exists in all cells) [22,23]. Partial trisomy is typically only of the 13q type, meaning the extra copy present is that of the long arm (q) of chromosome 13 [23]. Furthermore, partial trisomy 13q is characterized by the presence of distinct symptoms including microcephaly, holoprosencephaly, leukoencephalopathy, facial anomalies, and intellectual disability [24,25].

We report a case of bilateral MMS occurring in a 19-year-old patient, secondary to underlying partial trisomy 13q. To our knowledge, this is the first recorded case of the two syndromes coexisting in a patient in literature. We believe her partial trisomy 13q has undue influence on her prognosis and improvement, due to the associated oxidative stress and mitochondrial illnesses. This matter has never been discussed previously, making it imperative that further research is conducted to identify the possible consequences of trisomy 13 on MMS.

While little to no data exists on the association between trisomy 13 and MMS, it has been established that the pathogenic changes seen in trisomy 13 are a direct cause of oxidative stress, with the faulty genes being primarily involved in the redox balance regulation [26]. The primary pathogenesis of trisomy 13 involves mitochondrial dysfunction [11], while the pathogenesis of MMS involves progressive narrowing of the intracranial portion of the distal ICA and the initial proximal components of the MCA and anterior cerebral artery [3]. In an extensive study by Choi et al. the two were found to be linked; it was determined that the mitochondria of the ECFCs in MMS patients were functionally and morphologically defective and had increased levels of ROS compared to control groups [12]. ECFCs have been established to play a role in pathological angiogenesis, with several reports demonstrating an intimate relationship between dysfunctional ECFCs and moyamoya [12,27,28]. Choi's finding of dysfunctional mitochondria within ECFCs in moyamoya patients strongly suggests that MMS may be a mitochondrial disorder [12]. It is in this context that we believe the patient's partial trisomy 13q was in fact the causative agent behind her MMS, however more research is needed to elucidate the association between the two.

Furthermore, Alramadan et al. established that 73% of pediatric moyamoya patients who underwent EDAS had improved seizure outcomes after surgery. Six out of eleven patients also became seizure-free [29]. Furtado et al. report similar findings, determining that 39 out of 43 patients on follow-up after EDAS showed stable or improved clinical symptoms with 90% event-free status at last follow-up [30]. However, despite undergoing bilateral EDAS, our patient continues to suffer from quadriplegia, intractable seizures, and spasticity in all limbs. However, our patient's findings resemble those of a report by Ribacoba et al. describing a 33-year-old female with partial trisomy 13q22 who developed leukoencephalopathy and late-onset epilepsy and stroke, and eventually passed away from nocturnal status epilepticus [9]. Brogna et al. report similar findings in a 23-year-old male with partial 13q22-q34 trisomy, who also developed seizures and leukoencephalopathy [10]. These findings reaffirm that partial trisomy 13q has been associated with refractory epilepsy, as is the case in our patient. While it has been established that the association between full trisomy 13 and seizures [31] is owed to the presence of structural lesions [9], this has yet to be proven in patients with partial trisomy.

## 4 Conclusion

In conclusion, 13q14 and 13q31 are crucial chromosomal regions that may be associated with oxidative stress and mitochondrial abnormalities leading to the development of MMS. We present a case of partial trisomy 13q that led to mitochondrial disease and ECFC dysfunction, leading to the development of MMS. Unfortunately, despite bilateral EDAS, the patient's MMS continued to progress, resulting in refractory seizures and spastic quadriplegia. MMS should be suspected and investigated in all children presenting with stroke. Greater research is needed to delineate the clinical features of MMS refractory to EDAS. Partial trisomy 13 is a significant chromosomal abnormality and studies should aim to shed more light on its varying presentation and genetic expression and elucidate the mechanisms of epilepsy associated with chromosomal abnormalities, particularly in the context of underlying mitochondrial illnesses. The number of studies demonstrating how mitochondrial damage is implicated in trisomy disorders is limited and greater research must be conducted to establish preventative measures, such as early screening tests for moyamoya disease. Lastly, we urge clinicians to suspect underlying cytogenetic causes in all pediatric patients presenting with recurrent strokes and seizures, hasten diagnosis, and begin appropriate treatment before further disease progression.

## Ethical approval

Written informed consent was obtained from the patient's parents for publication of this case report and accompanying images. Patient anonymity is maintained throughout this manuscript.

## Declarations

### Author contribution statement

All authors listed have significantly contributed to the investigation, development and writing of this article.

### Funding statement

This research did not receive any specific grant from funding agencies in the public, commercial, or not-for-profit sectors.

### Data availability statement

Data associated with this study has been deposited at Mendeley Data, V1, https://doi.org/10.17632/7b8nf7gp3x.1.

### Declaration of interest's statement

The authors declare no competing interests.
